# Patient perspectives on BCMA-targeted therapies for multiple myeloma: a survey conducted in a patient advocacy group

**DOI:** 10.3389/frhs.2024.1354760

**Published:** 2024-04-24

**Authors:** Jay R. Hydren, Dee Lin, Nathan W. Sweeney, Bingcao Wu, Nina Kim, Saurabh Patel, Douglas W. Sborov, Jesus G. Berdeja, Larry D. Anderson, Stephen Huo, Jorge Arturo Hurtado Martínez, Jennifer M. Ahlstrom

**Affiliations:** ^1^ HealthTree Foundation, Lehi, UT, United States; ^2^ Janssen Scientific Affairs, LLC, Horsham, PA, United States; ^3^ Janssen US Oncology Medical Affairs, Horsham, PA, United States; ^4^ Huntsman Cancer Institute, University of Utah, Salt Lake City, UT, United States; ^5^ Sarah Cannon Research Institute and Tennessee Oncology, Nashville, TN, United States; ^6^ Myeloma, Waldenstrom’s, and Amyloidosis Program, Simmons Comprehensive Cancer Center, UT Southwestern Medical Center, Dallas, TX, United States

**Keywords:** multiple myeloma, T-cell redirected therapy, BCMA-targeted therapy, CAR T-cell therapy, bispecific antibody, patient perspectives

## Abstract

**Background:**

Advances in multiple myeloma (MM) treatment have shifted the therapeutic landscape. Understanding patients' perspectives can assist physicians in helping patients make informed decisions. This study aimed to understand the patient decision-making process and gain insights into patient perspectives on B-cell maturation antigen (BCMA)-targeted therapies for MM.

**Methods:**

An 18-question survey was completed by patients with MM enrolled in HealthTree® Cure Hub, an online portal helping patients with plasma cell dyscrasias navigate their disease.

**Results:**

From October 28, 2022, to January 12, 2023, 325 patients with MM participated in the survey. The mean age (standard deviation) of the respondents was 66 (8) years; 54% were female and 90% were White. Among 218 patients with complete clinical records in the database, the median (min, max) lines of therapy (LOT) was 2 (1,16). Among 61 (28%) patients who had received ≥4 LOTs, 55 (90%) were triple-class exposed. Of the 290 patients who responded to the question about openness to new therapies, 76 (26%) were open to trying a new therapy immediately and 125 (43%) wanted more information on safety and efficacy. Most respondents reported likely or very likely to try a BCMA CAR T-cell therapy (60%) or a bispecific antibody (74%) and some needed more information to decide (16% for CAR T-cell therapy and 13% for bispecific antibody). The most requested information included efficacy, side effects (SEs), eligibility, and administration process for both CAR T-cell and bispecific therapies. When 2 therapies with the same efficacy and duration of response were offered, 69% of respondents would prefer the therapy with a lower risk of severe SEs but requires continuous dosing with no treatment-free interval, and 31% preferred a therapy given once followed by a treatment-free interval but with a potentially higher risk of severe SEs. To receive an effective therapy, the top acceptable trade-offs included frequent monitoring of SEs and initiating a new therapy in a hospital setting, and the least acceptable compromise was caregiver burden.

**Conclusions:**

This study found a high level of openness in patients with MM to try BCMA-targeted therapies. Information on efficacy, safety, availability, and eligibility may assist patients on decision-making.

## 1 Introduction

Multiple myeloma (MM), a plasma cell malignancy, is the second most prevalent hematologic cancer with estimated 35,730 new cases and 12,590 deaths in the United States (US) in 2023 (1). The 5-year relative survival for MM in the US is approximately 60% (1, 2). Over the past 15 years, patient survival has improved because of the introduction of targeted therapies, including immunomodulatory drugs (IMiDs), proteasome inhibitors (PIs), and monoclonal antibodies targeting anti-CD38. Despite the advances in treatment, patients with MM eventually progress resulting in relapsed/refractory multiple myeloma (RRMM). Patients with RRMM often suffer from disease progression, experience a high disease burden and complications associated with prior therapies (3), and have reduced health-related quality of life (HRQoL). Studies have shown that further treatment options, along with prolongation of life expectancy and effectiveness of treatment, are of foremost importance to patients with MM (4). However, studies evaluating holistic care approaches also have reported the impact of side effects (SE) on treatment decision-making, and acknowledged that patients with MM have different definitions regarding efficacy (5–7).

The emergence of T-cell redirected therapy (TCRT) has markedly improved clinical outcomes in patients with heavily pretreated MM. Multiple potential targets in MM have been identified including the B-cell maturation antigen (BCMA; a tumor necrosis factor receptor superfamily member 17 surface protein found predominantly on mature B-lymphocytes and plasma cells but not in other normal cells), CD24, CD38, CD56, CD138, signaling lymphocytic activation molecule family member 7 (SLAMF7), programmed death-ligand 1 (PD-L1), G protein-coupled receptor class C group 5 member D (GPRC5D), and integrin beta7. At the time of writing this article, the FDA has approved 5 TCRTs, including 2 BCMA-targeted chimeric antigen receptor (CAR). CAR hasn't been defined before this. CAR T-cell products [idecabtagene vicleucel (8) and ciltacabtagene autoleucel (9)], 2 bispecific BCMA-directed CD3T-cell engagers [teclistamab (10) and elranatamab (11)], and 1 GPRC5D-targeted bispecific antibody [talquetamab (12)]. However, when the survey was conducted (October 28, 2022 to January 12, 2023), only 3 BCMA-targeted TCRTs were approved [i.e., ide-cel in 2021 (8), and cilta-cel (9) and teclistamab in 2022 (10)]; elranatamab and talquetamab were both approved in August 2023 after this survey had completed. This present study therefore focused on BCMA-targeted TCRTs, including CAR T-cell therapies and bispecific antibodies.

Both BCMA-targeted CAR T-cell therapies and bispecific antibodies have resulted in high response rates in patients with RRMM with overall response rate (ORR) in clinical trials ranging from 63% for teclistamab (13), to 73% for ide-cel (14) and 97% for cilta-cel (15), and are approved for the same patient population. However, the two classes of therapies have distinct characteristics and are associated with different benefits and shortcomings. As a single infusion product, CAR T-cell therapies require relatively shorter treatment time while offering a potentially long remission. However, during the study period only a limited number of treatment centers had the expertise and staffing to administer CAR T-cell therapies. Furthermore, CAR T-cell therapies are manufactured at special facilities and are associated with additional logistical and clinical challenges including lengthy manufacturing times, limited manufacturing lots, potential manufacturing failure, requirement for bridging therapy and lymphodepleting conditioning therapy prior to CAR T-cell infusion, lengthy post-infusion hospital stay, and specialized care. On the other hand, as “off-the-shelf” products, bispecific antibodies do not have manufacturing delays, do not need bridging therapies, and can be administered at more locations including academic treatment centers and some community hospitals. However, bispecific antibodies such as teclistamab (the only bispecific antibody approved during this study period) are administered until unacceptable toxicity or they stop working (i.e., disease progression). Other differences between CAR T-cell therapies and bispecific antibodies include SEs, adverse event (AE) monitoring, costs, and the need for a caregiver. All these differences can affect a patient's treatment decision, experience, and outcomes.

Given that BCMA-targeted CAR T-cell therapies and bispecific antibodies have become commercially available only in the past couple years, little is known about patients' knowledge, decision-making processes, and openness to these novel BCMA-targeted therapies. In previous qualitative research, 7 domains linked to patient decision-making were identified, including (1) treatment effectiveness, (2) treatment experience, (3) impact on daily life, (4) treatment type preference, (5) treatment features, (6) tolerability or SEs, and (7) mode of administration (5–7). The present study sought to extend this understanding by incorporating inferential statistics to quantify the influence of these domains, as well as 9 additional questions specifically related to BCMA-targeted CAR T-cell and bispecific therapies for MM. The additional questions encompass (1) treatment education, (2) treatment resources, (3) level of involvement in the treatment decision-making process, (4) impact on the patient's ability to care for themselves or others, (5) clinical trial decision-making with limited therapeutic knowledge, (6) qualification for treatment, (7) availability of novel treatments, (8) patient preference for specific novel treatments, and (9) exploration of additional topics beneficial in treatment decision-making. Taken together, the objectives of this study were to (1) understand patients' decision-making process, (2) gain insights into patients’ expectations and evaluation of the treatment options for MM, and (3) learn patients' understanding and perceptions of BCMA-targeted therapies. By understanding patient preferences, values, knowledge gaps, and willingness to try new therapies, healthcare providers can better support patients in making informed treatment decisions.

## 2 Materials and methods

### 2.1 Study design

This is a cross-sectional survey completed by patients with MM enrolled in HealthTree® Cure Hub (16–18), an online portal to help patients with plasma cell dyscrasias navigate their diseases. HealthTree® Cure Hub allows a patient to aggregate their medical data while receiving numerous portal benefits (i.e., personalized treatments, community groups, and research participation). Upon self-selected enrollment, the patient provides electronically signed consent to share their medical data, which include information regarding the patient's diagnosis, physician(s) and caregiver, health and fitness, treatment history, laboratory, imaging, and genetic results, and full health profile including demographics, health history, family history, and lifestyle, also known as the Data Dictionary. The Data Dictionary is refreshed at least once a year.

Patients provided online consents through the HealthTree® Cure Hub portal prior to the survey. The survey took place between October 28, 2022, and January 12, 2023. Survey responses were linked to existing data from HealthTree's Data Dictionary.

### 2.2 Patient population

All patients (>11,000) enrolled in the HealthTree® Cure Hub platform during the survey period (October 28, 2022 to January 12, 2023) were notified of the survey via e-mail. Adult patients (≥18 years of age) diagnosed with MM who were receiving treatment for MM in the US, could read and write English, consented to the study, and filled out the online survey were included in the analyses.

### 2.3 Survey instrument

The survey was developed by the research team (authors DL, BW, NK, SH, JH, JM, and JA). Each question was reviewed by author JH and a 5-member HealthTree Patient Advocacy Panel using a focus group discussion with a focus on clarity and narrow interpretation of each question and option, while emphasizing the need to ensure the language is accessible to minimally educated patients—a process aimed at identifying and addressing potential validity and reliability issues within the original draft of the survey. After these adjustments, the physician panel (authors DS, JB, and LA) reviewed the questions with a focus on scope, validity, and potential reliability issues based on their personal clinical and research experiences with BCMA-targeted CAR T-cell and bispecific antibody therapies for patients with RRMM. The feedback from the physician panel indicated that an integrative process to address missing domains, adjust the scope, or address potential validity or reliability concerns was not needed.

The 18-question patient online survey assessed patients' perspectives in 3 general domains: treatment decision-making process, patients' expectation and evaluation of MM treatment, and patient perspectives and information needs related to BCMA-targeted therapies including CAR T-cell therapy and bispecific antibodies (Supplementary Table S1). Content of the questionnaire is outlined in Table 1. Respondents were not required to answer all questions in the questionnaire. For some questions, respondents were asked to rank items listed for each question, but they were not required to rank all items. There were also some questions where respondents were asked to select all items that applied.

**Table 1:** The survey questionnaire topics^a^.

**Treatment decision-making process:**
• People involved in treatment decision-making, including HCPs and non-HCPs • Patients’ level of involvement in treatment decision-making • Patients’ mode of transportation and distance from treatment center
**Patients’ expectation and evaluation of MM treatment:**
• Factors that encourage one to consider changing treatment • Tradeoffs that the patient would be willing to make • Side effects that would make a patient choose not to receive a MM treatment • Challenges that the patient is facing regarding MM treatment • Openness to new therapies • Factors that are important when choosing MM treatment • Confidence level on other treatment options for MM if one relapses while on current treatment • Evaluation of supporting materials/programs to support MM treatment experience
**Patients’ perspectives and information needs related to BCMA-targeted therapies for MM:**
• Awareness of BCMA-targeted therapy and sources • Likelihood to try a CAR T-cell therapy if available • Likelihood to try a bispecific antibody if available • Needs of additional information on CAR T-cell therapy to support patient decision-making • Needs of additional information on bispecific antibodies to support patient decision-making

^a^ Complete survey questions are provided in Supplemental Material.

BCMA, B-cell maturation antigen; CAR, chimeric antigen receptor; HCP, healthcare providers; MM, multiple myeloma.

### 2.4 Data analysis

De-identified responses were analyzed for all respondents. All variables were analyzed descriptively and reported in aggregate. Mean and standard deviation (SD) were reported for continuous variables, whereas numbers and percentages were reported for categorical variables. A rank score was calculated as the sum of the inverse rank order by the respondent, then summed across the sample order data, and reported from the highest to the lowest.

Variations between subgroups of interest (i.e., newly diagnosed MM, RRMM, 1–3 lines of therapy [LOT], ≥4 prior LOT, and triple-class exposed [TCE, patients had been treated with at least an IMiD, a PI, and an anti-CD38 monoclonal antibody] plus ≥4 LOT) were evaluated by performing statistical analyses using standardized mean difference (SMD ≥ 0.1 indicating imbalance).

### 2.5 Compliance with ethical standards

The study protocol was approved by a WCG institutional review board (WCG IRB, Princeton, NJ, USA). All study participants provided written informed consent prior to participation in the survey.

## 3 Results

### 3.1 Patient characteristics

A total of 325 patients with MM who met the inclusion and exclusion criteria participated in the survey (Table 2). The mean (SD) age of the respondents was 66 (8) years, 54% (171/319) were female. The majority of the respondents identified themselves as White (228/253, 90%), followed by Black (15/253, 6%) and Other (9/253, 4%); 15% (37/245) were Hispanic. Among 246 respondents who answered the question about insurance, about half patients (129/246, 52%) reported having commercial insurance (44% through work and 8% through personal plan); most of the other half of patients had Medicare coverage (36% Medicare Part B, 35% Medicare Part A, 23% Medicare Part D, 19% Medigap/Medicare supplemental, and 14% Medicare Advantage), 2% had Medicaid, and 8% had other types of insurance (8%). A majority of the respondents (228/254, 90%) reported having some college, college completion, or above college level of education.

**Table 2:** Demographics, disease status, and treatment history of the patients with multiple myeloma who participated in the survey.

Demographics Total number of patients with MM surveyed (*N* = 325)	
Age (319 respondents, 98% of total surveyed)	
Mean age (SD), years	66 (8)
*N* (% of total respondents)	
Sex (319 respondents, 98% of total surveyed)	
Female	171 (54%)
Male	148 (46%)
Ethnicity (245 respondents, 75% of total surveyed)	
Hispanic or Latino	37 (15%)
Racial Background (253 respondents^a^, 78% of total surveyed)	
White (Original ancestry from Europe, Middle East, North Africa)	228 (90%)
Black or African American (Original ancestry from Africa)	15 (6%)
Other	9 (4%)
Type of Insurance (246 respondents,76% of total surveyed)	
Respondents asked to select all that apply	
Private commercial insurance through work	109 (44%)
Private commercial Insurance through personal plan	20 (8%)
Medicare Part B	88 (36%)
Medicare Part A	87 (35%)
Medicare Part D	57 (23%)
Medigap or Medicare supplemental	47 (19%)
Medicare Advantage	34 (14%)
Medicaid	5 (2%)
Other	20 (8%)
Highest level of education (254 respondents, 78% of total surveyed)	
College and above	192 (76%)
Some college or associate degree	36 (14%)
High school or below	25 (10%)
Other	1 (< 1%)
Participant Disease Status	
R-ISS Stage at Diagnosis (176 respondents, 54% of total surveyed)	
Stage I	50 (28%)
Stage II	65 (37%)
Stage III	42 (24%)
Unknown	19 (11%)
Treatment history	
LOT (218 respondents, 67% of total surveyed)	
Median LOT (min, max)	2 (1, 16)
1	71 (33%)
2	59 (27%)
3	27 (12%)
≥4 (4–16)	61 (28%)

LOT, lines of therapy; R-ISS, Revised International Staging System for Multiple Myeloma; SD, standard deviation.

^a^ Including 1 respondent who chose “I do not wish to provide this information.”

Among 176 patients who responded to the question regarding disease status at diagnosis, 37% (*n* = 65) had R-ISS Stage II, 28% (*n* = 50) had Stage I, and 24% (*n* = 42) had Stage III disease; 11% (*n* = 19) had unclassified disease. Among 218 patients who had complete clinical records in the database, the median (min, max) LOT was 2 (1,16); 33% (*n* = 71) patients had newly diagnosed MM and 39% (*n* = 86) had 2–3 LOT. Among the 61 patients who had at least 4 LOT, 90% were TCE.

### 3.2 Patient treatment decision-making

Among 325 patients responded to questions focused on treatment decision-making, 91% (*n* = 295) reported that they were involved in their treatment decision-making. Other individuals who were reported by respondents as being involved included myeloma specialists (64%), primary and consulting oncologists (60% and 15%, respectively), caregivers, other family members or friends (39%), as well as other healthcare professionals (nurses, 3%; primary care doctors, 3%; and pharmacists, 2%) (Table 3). More than half of the patients (59%, *n* = 190) reported that their doctor drove the conversation regarding treatment-decision but considered their goals and preferences, while 32% (*n* = 102) patients reported they were very involved in their treatment decision-making and led the conversation with their doctor (Table 3). Subgroup analyses showed that as patients progressed through lines of therapy, they became more involved in their treatment decision, were more concerned about SEs that may require supportive care, found dealing with SEs more challenging, were less confident that a treatment option would be available after their next relapse, and were more concerned that their health conditions would limit their treatment options (Supplementary Table S3).

**Table 3:** Patient treatment decision-making for multiple myeloma.

Total number of patients with MM surveyed (*N* = 325)	*n* (% of total respondents)
Individuals involved in MM treatment decision-making	
Respondents (325, 100% of total surveyed)	
Patient	295 (91%)
Myeloma specialist	208 (64%)
Primary oncologist/hematologist	195 (60%)
Caregiver, other family members, or friends	126 (39%)
Consulting oncologist/hematologist	48 (15%)
Nurse	11 (3%)
Primary care doctor	11 (3%)
Pharmacist	6 (2%)
Other	7 (2%)
Patient involvement in MM treatment decision-making	
Respondents (323, 99% of total surveyed)	
Very involved, my doctor drives the conversation but considers my goals and preferences with recommendations	190 (59%)
Very involved, I drive the conversation	102 (32%)
Somewhat involved, my doctor makes the decision and asked for my agreement	30 (9%)
Not so involved, I let my doctor make the decision	1 (<1%)
Patients’ primary mode of transportation to MM treatment center	
Respondents (323, 99% of total surveyed)	
Car	305 (94%)
Walk	7 (2%)
Plane	4 (1%)
Bus	3 (1%)
Uber/Lyft/Taxi	3 (1%)
Train	1 (<1%)
Patients’ travel time to MM treatment center	
Respondents (322, 99% of total surveyed)	
0–30 min	155 (48%)
30–60 min	93 (29%)
1–2 h	42 (13%)
2–4 h	23 (7%)
4–10 h	6 (2%)
More than 10 h	3 (1%)

A majority of the patients (94%, 305/323) reported that their primary mode of transportation to MM treatment centers was car (94%, 305/323), followed by walk (2%, 7/323), and other transportation modes (1% for plane, bus, and Uber/Lyft/Taxi, respectively, and <1% for train). Most patients (77%, 248/322) spent ≤1 h traveling to their MM treatment centers, 13% (42/322) spent 1–2 h, and 10% (33/322) spent >2 h (Table 3).

### 3.4 Patients' expectation and evaluation of new treatment for MM

When choosing MM treatment options, most patients (81%, 224/277) ranked a therapy's potential to extend life as the most important factor, followed by better quality of life (69%, 192/277), progression-free period (68%, 188/277), and SE tolerance (58%, 161/277). The majority of patients would consider changing current treatment for a new therapy if the new therapy could double the remission time (75%, 228/306), be fully covered by their insurance (73%, 224/306), or reduce the SE burden by half (64%, 195/306) (Supplementary Table S2).

**Figure 1:**
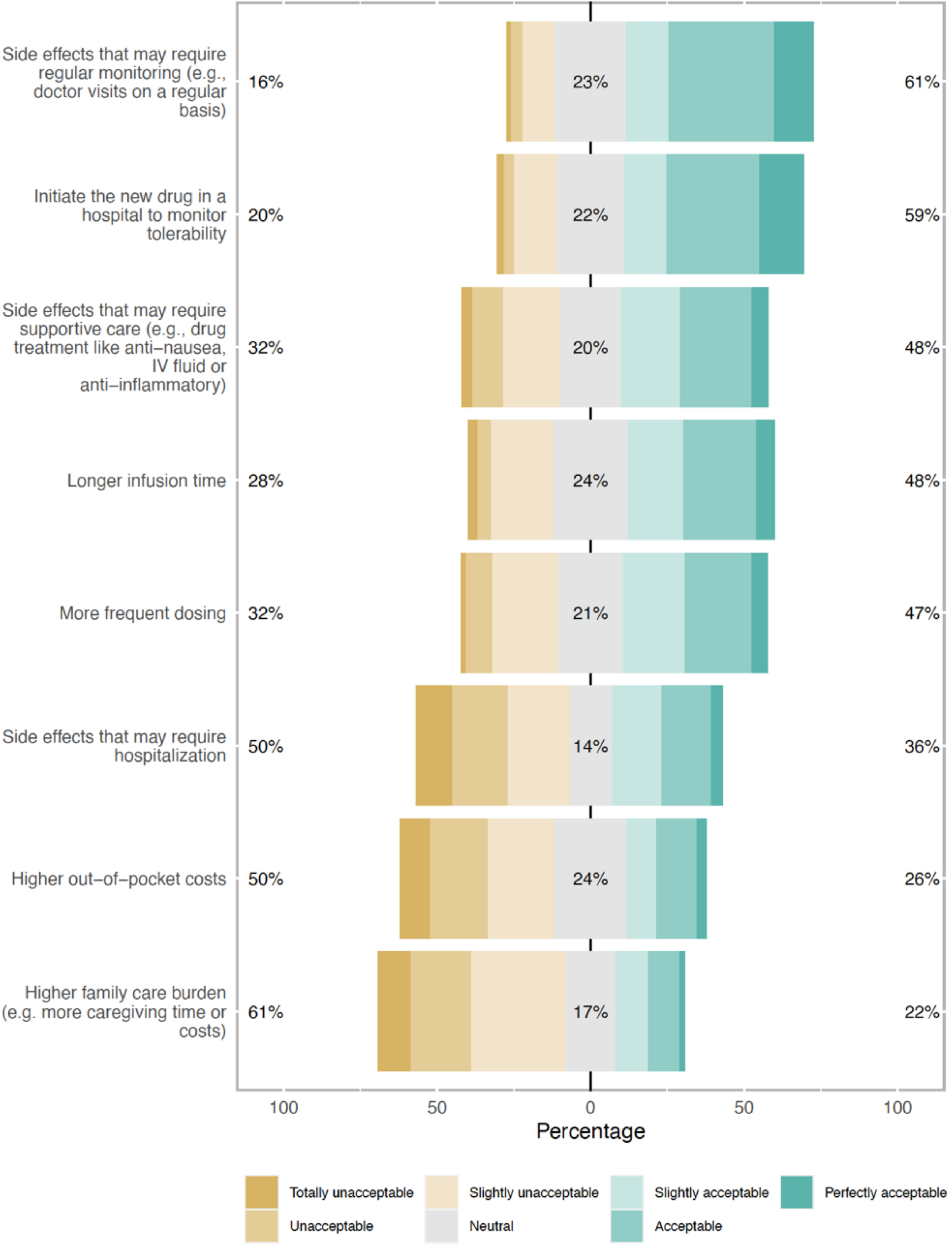
Types of trade-offs respondents were willing to make to receive multiple myeloma treatment that brings improved outcomes (% of total respondents).

To receive a treatment that brings improved outcomes, patients indicated willingness to accept certain “trade-offs.” The top acceptable “trade-offs” included frequent monitoring of SEs (61%) and initiating a new drug in a hospital setting (59%) (Figure 1). Patients were least willing to add more burden to their caregivers (61%). When asked about MM therapy preference, assuming the same efficacy and duration of response, 69% (201/292) of respondents reported preference for a therapy with lower risk of severe SEs but requiring continuous dosing with no treatment-free interval, as opposed to a therapy that is given once followed by a treatment-free interval but with a potentially higher risk of severe SEs (31%, 91/292) (Table 4).

**Table 4:** Survey results of patients’ perspectives on new therapies for multiple myeloma, including BCMA-targeted therapies.

Total number of patients with MM surveyed (*N* = 325)	*n* (% of total respondents)
Openness to new therapies	
Respondents (290, 89% of total surveyed)	
Very open, if eligible, I want to try as soon as possible	76 (26%)
Open, but would like to wait for more data on efficacy and safety	125 (43%)
Open, if other patients I know have tried it	5 (2%)
Open, if my health care provider recommends it	70 (24%)
I’m not interested in trying new therapy at the moment	12 (4%)
Not sure	2 (1%)
Awareness of BCMA targeted therapy and sources^a^	
Respondents (216, 67% of total surveyed)	
Yes, from online search	120 (56%)
Yes, from my healthcare providers	79 (37%)
Yes, from other resources	67 (31%)
Yes, from a clinical trial I participated in	21 (10%)
Yes, from family and friends	12 (6%)
Yes, from media advertisement	10 (5%)
No	17 (8%)
Likelihood of trying a CAR T-Cell therapy if available	
Respondents (198, 61% of total surveyed)	
I have already received one	16 (8%)
Very likely or Likely	119 (60%)
Neutral	21 (11%)
Very Unlikely or Unlikely	11 (6%)
I need more information to decide	31 (16%)
I have not heard of CAR T-Cell Therapy	0 (0%)
Likelihood of trying a bispecific antibody if available^a^	
Respondents (198, 61% of total surveyed)	
Very likely or Likely	146 (74%)
Neutral	22 (11%)
Unlikely	3 (2%)
I need more information to decide	26 (13%)
I have not heard of a bispecific antibody	1 (1%)
Additional information on CAR T-Cell therapy needed to support your decision for your MM treatment^b^	
Respondents (95, 29% of total surveyed)	
Efficacy—how well the therapy will provide me the desired clinical outcome	83 (87%)
Side effects	68 (72%)
Am I the right patient to receive it	61 (64%)
What is the administration process and procedure	51 (54%)
Costs to me	53 (56%)
Where I can receive it	50 (53%)
How will this therapy impact my family or caregivers	42 (44%)
How often I need to receive it	45 (47%)
How soon I can receive it	49 (52%)
Additional information on bispecific antibodies needed to support your decision for your MM treatment^b^	
Respondents (76, 23% of total surveyed)	
Efficacy—how well the therapy will provide me the desired clinical outcome	68 (89%)
Side effects	56 (74%)
Am I the right patient to receive it	48 (63%)
What is the administration process and procedure	41 (54%)
Costs to me	41 (54%)
How soon I can receive it	37 (49%)
How often I need to receive it	38 (50%)
Where I can receive it	38 (50%)
How will this therapy impact my family or caregivers	31 (41%)
Assuming the same efficacy and same duration of response, which therapy would you choose for your MM	
Respondents (292, 90% of total surveyed)	
“A therapy with less risk but requiring continuous dosing, no treatment-free interval”	201 (69%)
“A therapy that is given once followed by a treatment-free interval but with a potentially higher risk of severe side effects”	91 (31%)

BCMA, B-cell maturation antigen; CAR, chimeric antigen receptor; MM, multiple myeloma.

^a^ Respondents were asked to “select all that apply”.

^b^ Respondents were asked to “rank from the most to least important, ranking all is not required”.

**Figure 2:**
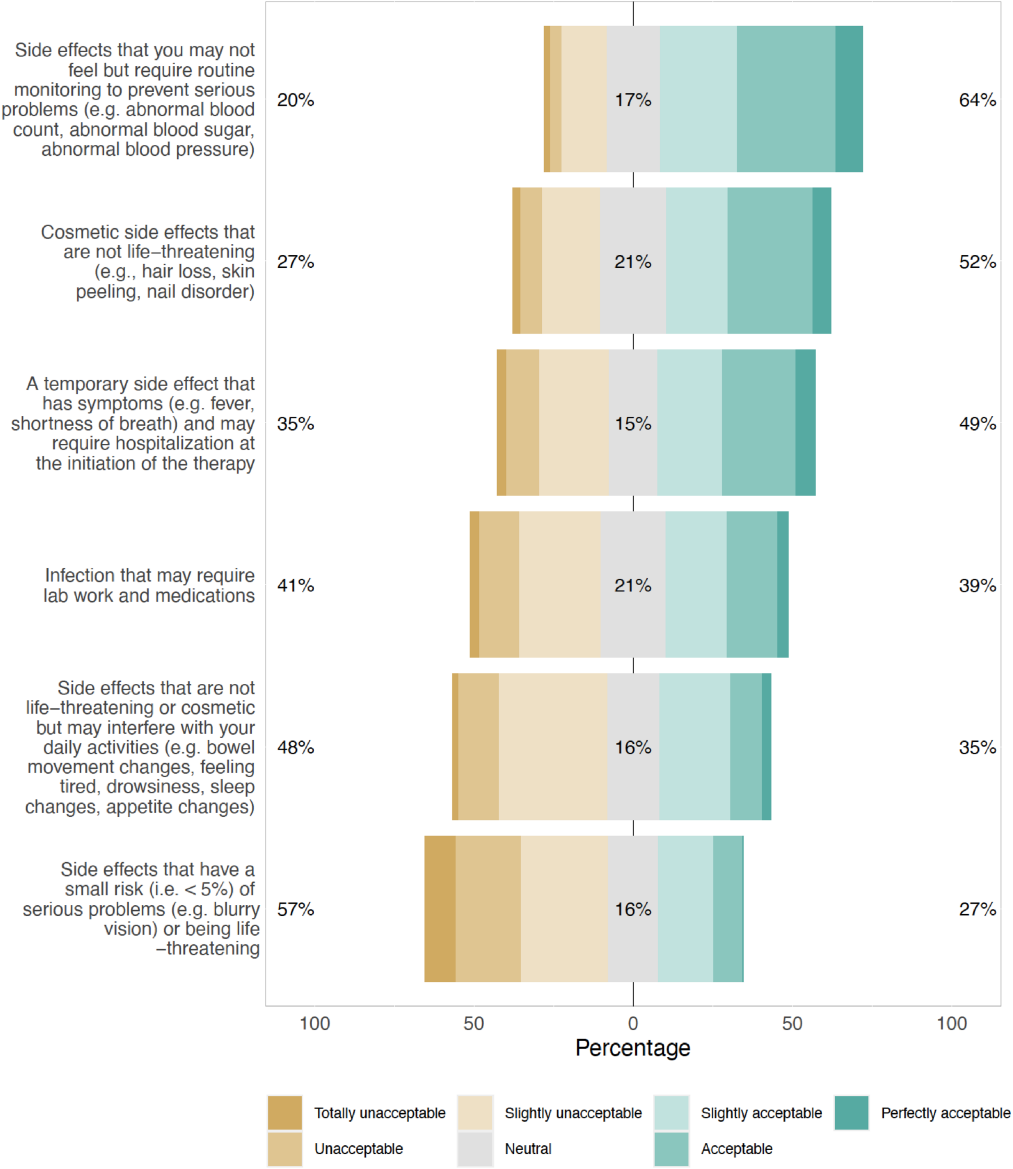
Type of side effects that would make respondents not want to receive a beneficial multiple myeloma treatment.

The most acceptable SEs reported by respondents were those that were asymptomatic but would need routine monitoring to prevent serious complications (64%), and those that were cosmetic but non-life-threatening (52%) (Figure 2). SEs that were rare but could cause serious problems or were life-threatening were least acceptable (27%) to the respondents. These findings were consistent across subgroups of patients with newly diagnosed (MM), RRMM, and different numbers of prior LOT, with no statistically significant differences observed. The top 4 MM treatment-related challenges patients reported experiencing were lack of effective treatment options (11%, 31/294), side effects (9%, 26/294), cost burden (6%, 19/294), and insurance coverage (6%, 19/294). Patients indicated a high level of confidence that there would be other treatment options available when their MM relapses [8.3 (2.2) on a scale of 0–10 with 0 being not confident at all and 10 being extremely confident] (Supplementary Table S2).

Of the 290 patients who responded to the question about their openness to trying a new therapy for MM, the majority (95%, 276/290) reported being open; 26% reported being open to trying right away, 43% reported being open but wanting more information about safety and efficacy, while 24% reported being open if their healthcare providers recommend it (Table 4). Patients identified the following materials and programs most helpful in supporting their MM treatment decision: patient-facing educational material on disease and retreatments (77%, 200/259), patient networks to connect with fellow patients (64%, 167/259), care navigation (49%, 127/259), and out-of-pocket cost support (49%, 126/259) (Supplementary Table S2).

### 3.5 Patients' perspectives and information needs related to BCMA-targeted therapies for MM, including CAR T-cell therapy and bispecific antibodies

Among 216 patients who responded to the question on awareness of BCMA-targeted therapies for MM, including CAR T-cell therapy and bispecific antibodies, 92% (199/216) patients reported having heard of BCMA-targeted therapies (Table 4) from various sources including online search (56%, 120/216) and healthcare providers (37%, 79/216). Only 8% (17/216) of the respondents reported being unaware of BCMA-targeted therapies. When asked about the likelihood of trying a BCMA-targeted therapy, most respondents reported likely or very likely (60% [119/198] and 74% [146/198] for CAR T-cell therapy and bispecific antibody, respectively), while 16% and 13% respondents indicated that they would need more information to decide (Table 4). 8% (16/198) of the respondents reported that they had already received a CAR T-cell therapy. A small percentage of patients reported that they were unlikely or very unlikely to try a CAR T-cell therapy (6%, 11/198) or bispecific antibody (2%, 3/198) (Table 4).

Regarding information needed to support decision-making for CAR T-cell therapy or bispecific antibodies, the most requested information for both therapies were efficacy, SEs, eligibility, administration process, and costs. “How soon can I receive it?” was ranked higher for bispecific therapy than CAR T-cell therapy, while “Where can I receive it?” and “How will this therapy impact my family or caregivers” were ranked higher for CAR T-cell therapy, relatively (Table 4). Additionally, patients also considered the potential impact on their family or caregivers important.

## 4 Discussion

The present study used an online patient survey to obtain patients' perspectives in 3 general areas: treatment decision-making process, patients' expectation and evaluation of MM treatment, and patient perspectives and information needs related to BCMA-targeted CAR T-cell therapies and bispecific antibodies. The study revealed a high-level of patient involvement and shared decision-making between patients and their doctors. Most participants of the survey reported that they were highly involved in their treatment decision-making, along with their oncologists/hematologists, caregivers, and other family members or friends. The majority of the respondents were open to trying new therapies and many of those patients wanted more information about a new therapy's efficacy and safety before making a decision, indicating a high unmet need for additional, effective, and safe treatment options among patients with RRMM.

Responses from this study showed that MM treatment decision-making is a complex process involving multiple considerations. When choosing MM treatment options, patients often consider a wide range of factors, particularly treatment efficacy, safety, and quality of life. Many patients reported that they would consider changing current treatment for a new therapy if the new therapy has a potential to double the remission time, is fully covered by their insurance, or markedly reduce SE burden, indicating patients' desire to improve quality of life and reduce cost and disease burden while living longer. This finding is consistent with other studies on patient perspectives conducted in the US (19) and other countries (4, 20–22). While efficacy has been consistently identified as the most important factor when choosing MM treatment options, a variety of other factors influence patients' decision-making, including quality of life and tolerability of SEs (19, 21–23).

Consistent with studies from other groups, our study showed that there is heterogeneity regarding how patients weigh and balance their considerations during the treatment decision-making process. While efficacy, safety, and quality of life were the most important deciding factors, the trade-offs that patients were willing to take differed depending on patient populations. For example, in 2 qualitative descriptive studies by Dombeck et al. and He et al., respectively (19, 20), patients expressed preference for more convenient treatment options; whereas in our survey more than half of the respondents were willing to accept certain inconvenience (e.g., regular AE monitoring, initiating a therapy in a hospital setting) for a treatment that could be clinically beneficial. Additionally, in the study by He et al, the patients with newly diagnosed MM and RRMM from the United Kingdom, France, and Germany did not identify financial impact as a burden whereas patients in our survey (US patients with RRMM) and patients in Parsons' study (Canadian patients with RRMM) identified costs of treatment and insurance coverage as a major consideration, which likely reflects the differences in healthcare systems and cost coverage between European countries, Canada, and the US.

Additionally, this survey highlighted the importance of patient education and peer support, particularly patient-facing educational materials on disease and treatment options and patient peer networks. Given the evolving treatment landscape for MM and the high level of patient involvement in decision-making, it is essential to provide patients with the information they need to help them make informed decisions.

To our knowledge, this is the first study on patient awareness and perspectives towards the use of novel BCMA-targeted therapies for MM and the type of information that is needed to assist patients in their decision-making process. Participants in this survey showed a high level of awareness of and would consider using BCMA-targeted therapies for MM, including CAR T-cell therapies and bispecific antibodies, and a majority of these patients reported a high level of likelihood to try these therapies, if offered. Efficacy, safety, eligibility, costs, and administration process were among the most important factors to the respondents when considering these treatment options. Regarding information needed to support decision-making, patients in this survey ranked “Where I can receive it” higher for CAR T-cell products and “How soon I can receive it” for bispecific antibodies, reflecting the different characteristics of the 2 types of novel therapies. Given that CAR T-cell therapies currently are available only at select centers, it is natural that patients would want to know the accessibility of a treatment center. On the other hand, as “off-the-shelf” products, commercial bispecific antibodies have become available only in the past year, which might explain why patients wanted to know how soon they could receive it, if eligible.

The assessment of caregiving support by patients was preliminarily investigated in this survey. While the impact on caregivers was anticipated to be influential for some patients, the surprising finding was that 40% (42/95) of patients considering CAR T-cell and 41% (31/76) of those contemplating bispecific antibodies expressed a desire for additional information on potential requirements. This was further evidenced by 39% (126/325) of patients reporting the involvement of “caregiver, other family member, or friends” in the decision-making process for MM treatment. Despite the constraints in wording and definition, this survey quantified the notable proportion of patients whose treatment decisions might be influenced by considerations of caregiver requirements after therapy initiation and discharge from treatment centers.

As the first known study assessing patients' perspectives on BCAM-targeted therapies, this study has a few additional strengths. First, patients' demographics, clinical characteristics, and treatment history have been collected regularly by HealthTree Cure Hub as part of their data dictionary. Availability of such data enabled rapid survey administration, data collection and analysis, and the richness of the data also allowed subgroup analysis to answer various research questions. Furthermore, this study had a relatively large sample size for this rare-disease population, and a targeted recruitment of minority populations on a non-profit patient education, empowerment, and engagement platform likely played a key role in reaching a diverse group of patients to ensure that their perspectives were represented in the study.

However, as a survey of patient perspectives on MM treatment, this study has the potential for biases such as response, social desirability, recall, and question formulation that are common in such study design. For instance, question 6 aimed to strike a balance between the necessity for a quantified benefit-risk analysis and the objective of creating a survey question set that was not overly burdensome, to reduce the risk of elevated survey attrition rates. Second, Patients who participated in the survey showed high self-reported levels of disease and treatment knowledge, which may be indicative of more informed patients. As the HealthTree platform is a comprehensive online multimedia education system taught by blood cancer specialists, this bias was anticipated. Internal research at HealthTree, comparing a survey conducted solely online with an identical survey carried out in a clinic, revealed differences in self-reported education levels for the survey topic before and after conducting coarsen exact matching to balance the data sets on known descriptive variables (unpublished data). However, this bias likely supports the overall findings of the study, as it documents the choices, behavior patterns, and experiences of a patient population possessing a working knowledge of a disease characterized by considerable complexities in chronic disease tracking and treatments. Third, 90% of the respondents in the present study were White even though MM incidence is twice as high among Black Americans as White Americans (24). Thus, the result of the current study may not be representative of the overall MM population, and further research is needed to identify effective and feasible outreach strategies to recruite statistically significant samples of these important subpopulations of interest. Additionally, patients participating in the survey were not required to respond to all questions or all the items in a question, resulting in incomplete data. Furthermore, for questions where the respondents were asked to rank items, they were not required to rank everything, likely resulting in high heterogeneity of reporting results in those cases. Finally, this study did not use any validated patient-reported outcome instruments. However, efforts were made to ensure the validity and reliability of the questionnaire through review by the HealthTree Patient Advocacy Panel and a physician panel (authors DSW, JGB, LDA), despite the absence of a test-retest analysis or convergent comparisons, as described in the methods section. Moreover, it is essential to note that 2 recent qualitative studies identified 7 domains related to treatment decisions (25, 26); this survey not only encompassed each of these reported constructs but also delved into 9 additional areas pertinent to the novel therapies of interest, resulting in a broad investigation involving a total of 16 quantified topics.

In summary, the emergence of novel targeted therapies has drastically changed the MM treatment paradigm. With increasing number of new therapies available, patients with MM have more options. For many patients, MM now can be managed like a chronic disease. However, optimizing the timing, sequence, and/or combination of available therapies remains a challenge. As patients become increasingly involved in their treatment decision-making process, understanding their perspectives and preferences is critical to optimize management strategies to ultimately improve patient treatment experience and outcome. Findings of this present study will benefit the development of a patient-centered treatment approach and shared decision-making between patients and physicians. It is important to note that priorities vary from patient to patient and may change as patients go through lines of therapy, as shown in our study and a recent survey by Ribbands et al. (27). With rapidly expanding availability of new therapies, surveys and studies like ours will continue to shape the treatment landscape and ultimately, redefine the patient-physician discussion.

To further understand the factors influencing the patient's treatment decision-making and to develop supportive strategies accordingly, we are conducting an additional 51-question survey covering 11 domains. Early results indicated that patients choose treatment options based on factors beyond clinical efficacy. Data analysis is ongoing.

## 5 Conclusions

Most respondents of this survey were highly involved and collaborated with their doctors during their treatment decision-making process. The most important factors influencing the respondents’ choice of treatment in order included efficacy, quality of life, duration of remission, and side effect tolerability. Patients were willing to accept certain tradeoffs, including regular adverse event monitoring, initiating a therapy in a hospital setting, and certain additional adverse events, for a treatment that could be clinically beneficial. The top supporting materials and programs that patients reported helpful are (in ranked order): patient-facing educational materials on disease and treatment, patient networks to connect with fellow patients, care navigation, and out-of-pocket cost support. Respondents of this survey showed a high level of awareness and openness to trying BCMA-targeted CAR T-cell and bispecific therapies, if offered. Information on efficacy, safety, availability, eligibility, and administration process of these new therapies may assist patients with their decision-making. Incorporating patients' goals, values, and preferences alongside clinical factors and other considerations may further optimize treatment decisions and improve patient outcomes.

## Data Availability

The datasets presented in this article are not readily available because HealthTree foundation does not send patient level data to outside organizations, however please contact corresponding author with specific questions or validation of data collected during this investigation. Requests to access the datasets should be directed to jay@healthtree.org.
