# Supplementary material for: Patient perspectives on BCMA-targeted therapies for multiple myeloma: a survey conducted in a patient advocacy group
Source: Front Health Serv. 2024 Apr 24;4:1354760. doi: 10.3389/frhs.2024.1354760 (PMC11078150; doi:10.3389/frhs.2024.1354760)
Supplement: Supplementary file 1 [file Datasheet1.docx]

# Supplemental Material

**Table S1. HealthTree Multiple Myeloma Patient Survey Questions**

| **The purpose of these next questions is to understand your treatment decision process.** |
| --- |
| 1. Who is involved in the decision-making for your multiple myeloma treatment? [Select all that apply]    1. Self    2. Primary Oncologist / Hematologist    3. Consulting Oncologist / Hematologist    4. Myeloma Specialist    5. Primary care doctor    6. Nurse    7. Pharmacist    8. Caregiver, other family members or friends    9. Other______ |
| 1. How involved are you in your treatment decision-making for multiple myeloma?    1. Very involved, I drive the conversation    2. Very involved, my doctor drives the conversation but considers my goals and preferences with recommendations    3. Somewhat involved, my doctor makes the decision and asked for my agreement    4. Somewhat involved, my caregiver drives the decision-making    5. Not so involved, I let my doctor make the decision    6. Other______ |
| 1. What is your primary mode of transportation to your myeloma treatment center?    1. Walk    2. Car    3. Uber/Lyft/Taxi    4. Bus    5. Train    6. Plane |
| 1. How far do you live from your myeloma treatment center (travel time)    1. 0-30 minutes    2. 30-60 minutes    3. 1 – 2 hours    4. 2 – 4 hours    5. 5-10 hours    6. Over 10 hours |
| **These next questions are about your expectation and evaluation of your treatment for multiple myeloma.** |
| 1. What is most important to you when choosing your multiple myeloma treatments? [Rank from the most to the least important]    1. Longer life    2. Longer time until disease progression or starting a new therapy    3. Better quality of life    4. Side effects are tolerable    5. Treatment-free period    6. Less frequent treatment or easier treatment    7. Cost of treatment (e.g., co-pay, travel, work productivity)    8. Burden to the family (e.g., family caregiving) |
| 1. Think about your current or most recent multiple myeloma treatment. What factors would encourage you to consider changing your current treatment for a new treatment? Please evaluate each factor individually [Likelihood Likert scale for each]    1. If the new treatment has 50% or longer time in remission (e.g., your remission is increased from 1 year to 1 and half years)    2. If the new treatment provides AT LEAST 2 times better remission (e.g., increase from 1 year to at least 2 years)    3. If the new treatment cuts the risk of side effects in half    4. If the same side effect is less severe (e.g., a severe headache becomes a mild-moderate headache)    5. If the new treatment is dosed less frequently (e.g., once every week becomes once every other week)    6. If the new treatment changed from an intravenous (IV) administration to subcutaneous (SC) shot    7. If the new treatment’s out-of-pocket cost to me is significantly lower    8. If the new treatment is fully covered by the insurance and does not cost anything to me |
| 1. In order to receive a treatment that brings improved treatment outcomes for your multiple myeloma, what are some “tradeoffs” that you would be willing to make? [Likelihood Likert scale on each of the following]    1. Side effects that may require hospitalization    2. Side effects that may require regular monitoring (e.g., doctor visits on a regular basis)    3. Side effects that may require supportive care (e.g., drug treatment like anti-nausea, IV fluid or anti-inflammatory)    4. More frequent dosing    5. Longer infusion time    6. Higher out-of-pocket costs    7. Initiate the new drug in a hospital to monitor tolerability    8. Higher family care burden (e.g., more caregiving time or costs) |
| 1. Assuming the same efficacy and same duration of response, which therapy below would you choose for your multiple myeloma?    1. A therapy that is given once followed by a treatment-free interval but with potential higher risk of severe side effects    2. A therapy with less risk but requiring continuous dosing, no treatment-free interval |
| 1. What types of side effects would make you NOT want to receive a multiple myeloma treatment that may be beneficial to you? [Likelihood Likert scale for each choice]    1. Cosmetic side effects that are not life-threatening (e.g., hair loss, skin peeling, nail disorder)    2. Infection that may require lab work and medications    3. Side effects that are not life-threatening or cosmetic but may interfere with your daily activities (e.g., bowl movement changes, feeling tired, drowsiness, sleep changes, appetite changes)    4. Side effects that you may not feel but require routine monitoring to prevent serious problems (e.g., abnormal blood count, abnormal blood sugar, abnormal blood pressure)    5. Side effects that have a small risk (i.e., < 5%) of serious problems (e.g., blurry vision or blindness) or being life-threatening    6. A temporary side effect that has symptoms (e.g., fever, shortness of breath) and may require hospitalization at the initiation of the therapy |
| 1. What are some challenges you are currently facing regarding your multiple myeloma treatments? [Severity Likert scale for each choice]    1. Lack of effective treatment options    2. Health conditions that limit treatment options (e.g., age, comorbidities)    3. Side effects    4. Inconvenient dosing schedule    5. Cost burden    6. Insurance coverage    7. Lack of social support    8. Other_____ |
| 1. On a scale from 0 to 10, how confident are you that there are other treatment options for your multiple myeloma if you relapse while on your current treatment?   [Scale from 0 to 10, 0 being not confident at all, 10 being extremely confident] |
| 1. How open are you to try a new therapy for your multiple myeloma if one becomes available?    1. Very open, if eligible, I want to try as soon as possible    2. Open, but would like to wait for more data on efficacy and safety    3. Open, if other patients I know have tried it    4. Open, if my health care provider recommends it    5. I’m not interested in trying new therapy at the moment    6. Not sure |
| 1. What type of supporting materials / programs are the most helpful to support your multiple myeloma treatment experience? [Rank from the most to the least helpful]    1. Patient network to connect with patients like me    2. Out-of-pocket cost support    3. Patient-facing educational materials on disease and treatments    4. Medical equipment    5. Caregiver support (e.g., resources for caregivers)    6. Transportation, lodging    7. Mental health support    8. Care navigation (e.g., doctor locator, care referral)    9. Other ________ |
| **These next questions are to estimate your understanding and perception of current or upcoming new therapies for multiple myeloma** |
| 1. Have you heard about the B cell maturation antigen (BCMA) targeted therapy for multiple myeloma? Some examples include chimeric antigen receptor T-cell therapy (CAR T-cell) and bispecific antibodies [select all that applied]    1. Yes, from my healthcare providers    2. Yes, from online search    3. Yes, from media advertisement    4. Yes, from family and friends    5. Yes, from other resources    6. Yes, from a clinical trial that I participated in    7. No (skip the next questions) |
| 1. Based on the information you have on CAR T-cell therapy, if it’s available to you, how likely would you like to try it for your multiple myeloma?    1. Very likely    2. Likely    3. Neutral    4. Unlikely    5. Very Unlikely    6. I need more information to decide    7. I have already received one (skip the next question)    8. I have not heard of a CAR T-cell (skip the next question) |
| 1. What additional information on CAR T-cell therapy do you need to support your decision-making for your multiple myeloma treatment? [Rank from the most to the least helpful; j will not be included for ranking; if j is selected, no need to rank the rest]    1. Efficacy – how well the therapy will provide me the desired clinical outcome    2. Side effects    3. Costs to me    4. How soon I can receive it    5. Where I can receive it    6. How often I need to receive it    7. Am I the right patient to receive it    8. How will this therapy impact my family or caregivers    9. What is the administration process and procedure    10. No additional information needed    11. Other__________ |
| 1. Based on the information you have so far on bispecific antibodies, if it’s available to you, how likely would you like to try it for your multiple myeloma?    1. Very likely    2. Likely    3. Neutral    4. Unlikely    5. Very Unlikely    6. I need more information to decide    7. I have not heard of a bispecific antibody (skip the next question) |
| 1. What additional information on bispecific antibodies do you need to support your decision making for your multiple myeloma treatment? [Rank from the most to the least helpful; j will not be included for ranking; if j is selected, no need to rank the rest]    1. Efficacy – how well the therapy will provide me the desired clinical outcome    2. Side effects    3. Costs to me    4. How soon I can receive it    5. Where can I receive it    6. How often I need to receive it    7. Am I the right patient to receive it    8. How will this therapy impact my family or caregivers    9. What is the administration process and procedure    10. No additional information needed    11. Other__________ |

**Table S2. Summary of HealthTree Multiple Myeloma Patient Survey Responses**

## Q1. Who is involved in the decision-making for your multiple myeloma treatment?

#### (Select all that apply)

responses / none-responses / total surveyed 325 / 0 / 325

**Responses**

Self 295 (91%)

Myeloma Specialist 208 (64%)

Primary Oncologist / Hematologist 195 (60%)

Caregiver, other family members, or friends 126 (39%)

Consulting Oncologist / Hematologist 48 (15%)

Nurse 11 (3%)

Primary care doctor 11 (3%)

Pharmacist 6 (2%)

Other 7 (2%)

#### Other (open responses, alphabetically listed)

responses: 7

"Cardiologist "

"My HealthTree Coaches and Social worker "

"National Institute for Clinical Excellence (UK)" "Spouse"

"wife"

"nurse practioner"

## Q2. How involved are you in your treatment decision-making for multiple myeloma?

#### (select one)

responses / none-responses / total surveyed 323 / 2 / 325

**Responses**

Very involved, I drive the conversation 102 (32%)

Somewhat involved, my doctor makes the decision and asked for my agreement 30 (9%)

Not so involved, I let my doctor make the decision 1 (<1 %)

Very involved, my doctor drives the conversation but considers my goals and 190 (59%)

preferences with recommendations

## Q3. What is your primary mode of transportation to your myeloma treatment center?

responses / none-responses / total surveyed 323 / 2 / 325

#### Responses

Car 305 (94%)

Walk 7 (2%)

Plane 4 (1%)

Bus 3 (1%)

Uber/Lyft/Taxi 3 (1%)

Train 1 (<1%)

## Q4. How far do you live from your myeloma treatment center (travel time)?

#### (Select one)

responses / none-responses / total surveyed 322 / 3 / 325

**Responses**

0-30 minutes 155 (48%)

30-60 minutes 93 (29%)

Between 1 - 2 hours 42 (13%)

Between 2 - 4 hours 23 (7%)

Between 4 - 10 hours 6 (2%)

Over 10 hours 3 (1%)

##

## Q5. What is most important to you when choosing your multiple myeloma treatments?

#### (Rank from the most to the least important, ranking all is not required)

responses / none-responses / total surveyed 277 / 48 / 325

**Responses**

n (%) Ranking Score

Better quality of life 192 (69%) 1127

Longer time until disease progression or starting a new therapy 188 (68%) 1044

Longer life 224 (81%) 1355

Side effects are tolerable 161 (58%) 895

Less frequent treatment or easier treatment 123 (44%) 603

Treatment-free period 117 (42%) 498

Burden to the family (e.g. family caregiving) 109 (39%) 484

Cost of treatment (e.g. co-pay, travel, work productivity) 106 (38%) 456

*Note:*

Ranking Score is the sum of the inverse rank order by the respondent then summed across the sample. Respondents did not need to rank all question options.

## Q6. Think about your current or most recent multiple myeloma treatment. What factors would encourage you to consider changing your current treatment for a new treatment? Please evaluate each factor individually [Likelihood Likert scale for each]

responses / financially impacted / none-responses / total surveyed 306 / 19 / 325

#### If the new treatment has 50% or longer time in remission (e.g., your remission is increased from 1 year to 1 and half years)

Not at all encouraging 11 (4%)

Slightly encouraging 45 (15%)

Somewhat encouraging 107 (35%)

Very encouraging 91 (30%)

Extremely encouraging 46 (15%)

#### If the new treatment provides AT LEAST 2 times better remission (e.g., increase from 1 year to at least 2 years)

Not at all encouraging 4 (1%)

Slightly encouraging 15 (5%)

Somewhat encouraging 55 (18%)

Very encouraging 116 (38%)

Extremely encouraging 112 (37%)

#### If the new treatment cuts the risk of side effects in half

Not at all encouraging 4 (1%)

Slightly encouraging 32 (10%)

Somewhat encouraging 68 (22%)

Very encouraging 121 (40%)

Extremely encouraging 74 (24%)

#### If the same side effect is less severe (e.g., a severe headache becomes a mild-moderate headache)

Not at all encouraging 9 (3%)

Slightly encouraging 47 (15%)

Somewhat encouraging 100 (33%)

Very encouraging 103 (34%)

Extremely encouraging 39 (13%)

#### If the new treatment is dosed less frequently (e.g., once every week becomes once every other week)

Not at all encouraging 9 (3%)

Slightly encouraging 39 (13%)

Somewhat encouraging 87 (28%)

Very encouraging 120 (39%)

Extremely encouraging 46 (15%)

#### If the new treatment changed from an intravenous (IV) administration to subcutaneous (SC) shot

Not at all encouraging 24 (8%)

Slightly encouraging 41 (13%)

Somewhat encouraging 72 (24%)

Very encouraging 93 (30%)

Extremely encouraging 70 (23%)

#### If the new treatment’s out-of-pocket cost to me is significantly lower

Not at all encouraging 30 (10%)

Slightly encouraging 41 (13%)

Somewhat encouraging 60 (20%)

Very encouraging 98 (32%)

Extremely encouraging 70 (23%)

#### If the new treatment is fully covered by the insurance and does not cost anything to me

Not at all encouraging 21 (7%)

Slightly encouraging 23 (8%)

Somewhat encouraging 33 (11%)

Very encouraging 82 (27%)

Extremely encouraging 142 (46%)

## Q7. In order to receive a treatment that brings improved treatment outcomes for your multiple myeloma, what are some “tradeoffs” that you would be willing to make? [Likelihood Likert scale on each of the following]

responses / financially impacted / none-responses / total surveyed 298 / 27 / 325

#### Side effects that may require hospitalization

Totally unacceptable 35 (12%)

Unacceptable 53 (18%)

Slightly unacceptable 60 (20%)

Neutral 41 (14%)

Slightly acceptable 48 (16%)

Acceptable 48 (16%)

Perfectly acceptable 11 (4%)

#### Side effects that may require regular monitoring (e.g., doctor visits on a regular basis)

Totally unacceptable 4 (1%)

Unacceptable 11 (4%)

Slightly unacceptable 32 (11%)

Neutral 69 (23%)

Slightly acceptable 41 (14%)

Acceptable 102 (34%)

Perfectly acceptable 38 (13%)

#### Side effects that may require supportive care (e.g., drug treatment like anti-nausea, IV fluid or anti-inflammatory)

Totally unacceptable 10 (3%)

Unacceptable 30 (10%)

Slightly unacceptable 55 (18%)

Neutral 59 (20%)

Slightly acceptable 57 (19%)

Acceptable 69 (23%)

Perfectly acceptable 16 (5%)

#### More frequent dosing

Totally unacceptable 4 (1%)

Unacceptable 25 (8%)

Slightly unacceptable 63 (21%)

Neutral 62 (21%)

Slightly acceptable 58 (19%)

Acceptable 64 (21%)

Perfectly acceptable 15 (5%)

#### Longer infusion time

Totally unacceptable 9 (3%)

Unacceptable 13 (4%)

Slightly unacceptable 60 (20%)

Neutral 72 (24%)

Slightly acceptable 53 (18%)

Acceptable 70 (23%)

Perfectly acceptable 18 (6%)

#### Higher out-of-pocket costs

Totally unacceptable 28 (9%)

Unacceptable 54 (18%)

Slightly unacceptable 63 (21%)

Neutral 68 (23%)

Slightly acceptable 28 (9%)

Acceptable 38 (13%)

Perfectly acceptable 9 (3%)

#### Initiate the new drug in a hospital to monitor tolerability

Totally unacceptable 6 (2%)

Unacceptable 10 (3%)

Slightly unacceptable 42 (14%)

Neutral 65 (22%)

Slightly acceptable 41 (14%)

Perfectly acceptable 43 (14%)

Acceptable 90 (30%)

#### Higher family care burden (e.g. more caregiving time or costs)

Totally unacceptable 31 (10%)

Unacceptable 58 (19%)

Slightly unacceptable 91 (31%)

Neutral 49 (16%)

Slightly acceptable 31 (10%)

Acceptable 30 (10%)

Perfectly acceptable 5 (2%)

## Q8. Assuming the same efficacy and same duration of response, which therapy below would you choose for your multiple myeloma?

responses / none-responses / total surveyed 292 / 33 / 325

#### Responses

"A therapy with less risk but requiring continuous dosing, no treatment-free interval" 201 (69%)

"A therapy that is given once followed by a treatment-free interval but with a potentially 91 (31%)

higher risk of severe side effects"

## Q9. What types of side effects would make you NOT want to receive a multiple myeloma treatment that may be beneficial to you? [Likelihood Likert scale for each choice]

responses / financially impacted / none-responses / total surveyed 298 / 27 / 325

#### Cosmetic side effects that are not life-threatening (e.g., hair loss, skin peeling, nail disorder)

Totally unacceptable 7 (2%)

Unacceptable 20 (7%)

Slightly unacceptable 54 (18%)

Neutral 62 (21%)

Slightly acceptable 57 (19%)

Perfectly acceptable 17 (6%)

Acceptable 79 (27%)

#### Infection that may require lab work and medications

Totally unacceptable 8 (3%)

Unacceptable 37 (12%)

Slightly unacceptable 76 (26%)

Neutral 61 (20%)

Slightly acceptable 57 (19%)

Acceptable 47 (16%)

Perfectly acceptable 10 (3%)

#### Side effects that are not life-threatening or cosmetic but may interfere with your daily activities (e.g. bowel movement changes, feeling tired, drowsiness, sleep changes, appetite changes)

Totally unacceptable 5 (2%)

Unacceptable 38 (13%)

Slightly unacceptable 101 (34%)

Neutral 49 (16%)

Slightly acceptable 66 (22%)

Acceptable 30 (10%)

Perfectly acceptable 8 (3%)

#### Side effects that you may not feel but require routine monitoring to prevent serious problems (e.g. abnormal blood count, abnormal blood sugar, abnormal blood pressure)

Totally unacceptable 5 (2%)

Unacceptable 11 (4%)

Slightly unacceptable 42 (14%)

Neutral 50 (17%)

Acceptable 92 (31%)

Slightly acceptable 72 (24%)

Perfectly acceptable 25 (8%)

#### Side effects that have a small risk (i.e. < 5%) of serious problems (e.g. blurry vision or blindness) or being life-threatening

Totally unacceptable 28 (9%)

Unacceptable 61 (20%)

Slightly unacceptable 81 (27%)

Neutral 47 (16%)

Slightly acceptable 51 (17%)

Acceptable 27 (9%)

Perfectly acceptable 1 (0%)

#### A temporary side effect that has symptoms (e.g. fever, shortness of breath) and may require hospitalization at the initiation of the therapy

Totally unacceptable 8 (3%)

Unacceptable 31 (10%)

Slightly unacceptable 65 (22%)

Neutral 46 (15%)

Slightly acceptable 60 (20%)

Acceptable 69 (23%)

Perfectly acceptable 18 (6%)

## Q10. What are some challenges you are currently facing regarding your multiple myeloma treatments? [Severity Likert scale for each choice]

responses / financially impacted / none-responses / total surveyed 294 / 31 / 325

#### Lack of effective treatment options

Extremely challenging 6 (2%)

Very challenging 25 (9%)

Somewhat challenging 44 (15%)

Slightly challenging 53 (18%)

Not at all challenging 163 (55%)

#### Health conditions that limit treatment options (e.g., age, comorbidities)

Extremely challenging 3 (1%)

Very challenging 9 (3%)

Somewhat challenging 33 (11%)

Slightly challenging 75 (26%)

Not at all challenging 173 (59%)

#### Side effects

Extremely challenging 2 (1%)

Very challenging 24 (8%)

Somewhat challenging 79 (27%)

Slightly challenging 113 (38%)

Not at all challenging 73 (25%)

#### Insurance coverage

Extremely challenging 8 (3%)

Very challenging 11 (4%)

Somewhat challenging 28 (10%)

Slightly challenging 60 (20%)

Not at all challenging 186 (63%)

#### Cost burden

Extremely challenging 10 (3%)

Very challenging 9 (3%)

Somewhat challenging 31 (11%)

Slightly challenging 62 (21%)

Not at all challenging 179 (61%)

#### Inconvenient dosing schedule

Extremely challenging 2 (1%)

Very challenging 9 (3%)

Somewhat challenging 35 (12%)

Slightly challenging 59 (20%)

Not at all challenging 188 (64%)

#### Lack of social support

Extremely challenging 1 (<1%)

Very challenging 9 (3%)

Somewhat challenging 22 (7%)

Slightly challenging 42 (14%)

Not at all challenging 214 (73%)

#### Other

Extremely challenging 7 (2%)

Very challenging 9 (3%)

Somewhat challenging 9 (3%)

Slightly challenging 16 (5%)

Not at all challenging 82 (28%)

## Q11. On a scale from 0 to 10, how confident are you that there are other treatment options for your multiple myeloma if you relapse while on your current treatment?

#### [Scale from 0 to 10, 0 being not confident at all, 10 being extremely confident]

**
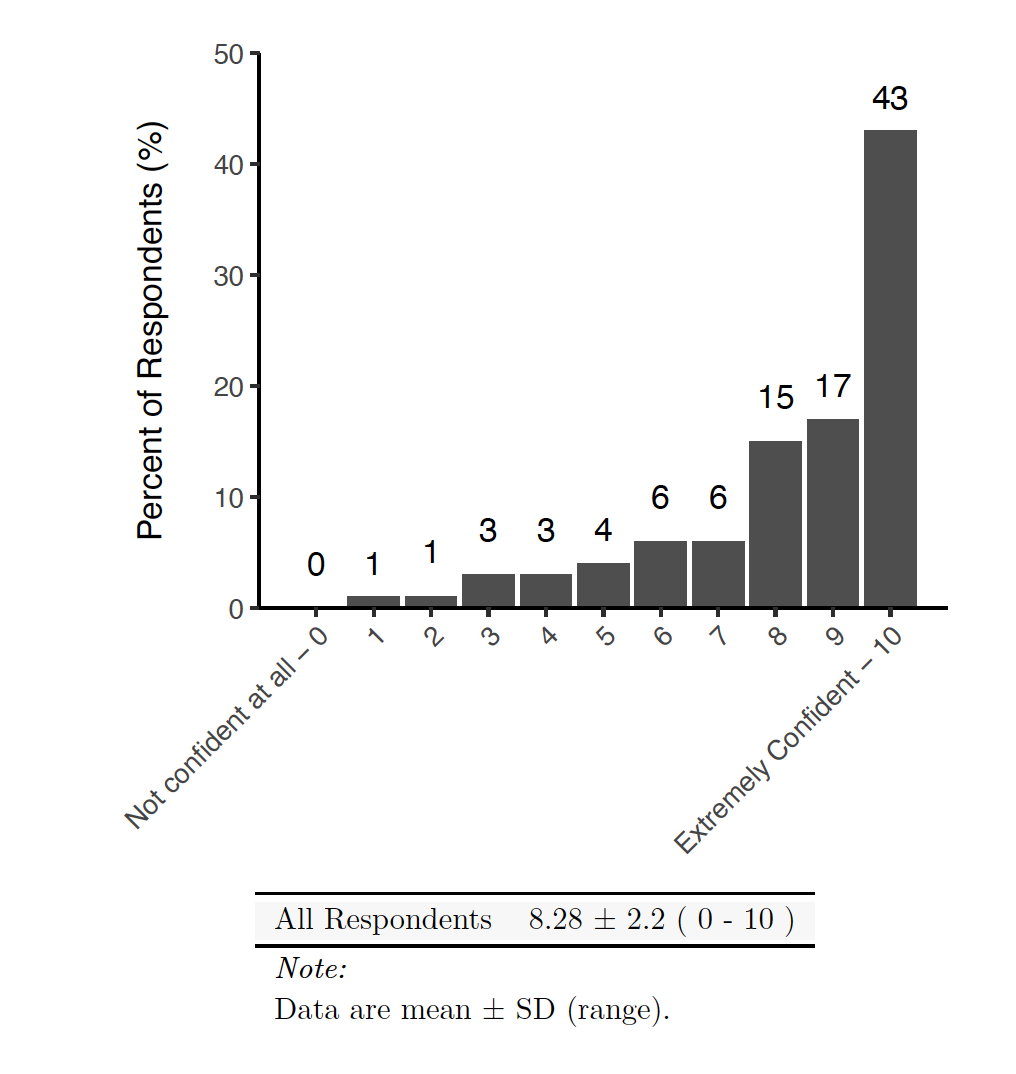
**

## Q12. How open are you to try a new therapy for your multiple myeloma if one becomes available?

#### (Select one)

responses / none-responses / total surveyed 290 / 35 / 325

**Responses**

Very open, if eligible, I want to try as soon as possible 76 (26%)

Open, but would like to wait for more data on efficacy and safety 125 (43%)

Open, if other patients I know have tried it 5 (2%)

Open, if my health care provider recommends it 70 (24%)

I’m not interested in trying new therapy at the moment 12 (4%)

Not sure 2 (1%)

## Q13. What type of supporting materials / programs are the most helpful to support your multiple myeloma treatment experience?

#### (Rank from the most to the least important, ranking all is not required)

n (%) Ranking Score

responses / none-responses / total surveyed 259 / 66 / 325

#### Responses

Patient network to connect with patients like me 167 (64%) 1037

Care navigation (e.g.,doctor locator, care referral) 127 (49%) 686

Patient-facing educational materials on disease and treatments 200 (77%) 1274

Out-of-pocket cost support 126 (49%) 686

Caregiver support (e.g., resources for caregivers) 102 (39%) 530

Transportation, lodging 95 (37%) 466

Medical equipment 82 (32%) 365

Mental health support 98 (38%) 436

*Note:*

Ranking Score is the sum of the inverse rank order by the respondent then summed across the sample. Respondents did not need to rank all question options.

#### Other (open responses)

responses: 72

## Q14. Have you heard about the B cell maturation antigen (BCMA) targeted therapy for multiple myeloma? Some examples include chimeric antigen receptor T-cell therapy (CAR T-Cell) and bispecific antibodies

#### (Select all that apply)

responses / none-responses / total surveyed 216 / 109 / 325

**Responses**

Yes, from online search 120 (56%)

Yes, from my healthcare providers 79 (37%)

Yes, from other resources 67 (31%)

Yes, from a clinical trial that I participated in 21 (10%)

Yes, from family and friends 12 (6%)

Yes, from media advertisement 10 (5%)

No 17 (8%)

## Q15. Based on the information you have on CAR T-Cell therapy, if it’s available to you, how likely would you like to try it for your multiple myeloma?

#### (Select one)

responses / none-responses / total surveyed 198 / 127 / 325

**Responses**

I have already received one 16 (8%)

Very likely 62 (31%)

Likely 57 (29%)

Neutral 21 (11%)

Unlikely 4 (2%)

Very Unlikely 7 (4%)

I need more information to decide 31 (16%)

Note: No respondents selected “I have not heard of CAR T-Cell.” There are 19 patients self-reporting having had CAR T-Cell therapy in this question, however there is 1 patient with CAR T-Cell in their medical records on HealthTree Cure Hub at the time the survey was conducted in this sample set (Table 2). There is a delay in when health records are validated, so it is unclear how many of the 19 patients have had CAR T-Cell or are in the process if having it at the time of this survey.

## Q16. What additional information on CAR T-Cell therapy do you need to support your decision-making for your multiple myeloma treatment?

#### (Rank from the most to the least important, ranking all is not required)

n (%) Ranking Score

responses / none-responses / total surveyed 95 / 230 / 325

#### Responses

Side effects 68 (72%) 355

Am I the right patient to receive it 61 (64%) 313

Efficacy – how well the therapy will provide me the desired clinical outcome 83 (87%) 457

What is the administration process and procedure 51 (54%) 238

Costs to me 53 (56%) 236

Where I can receive it 50 (53%) 221

How will this therapy impact my family or caregivers 42 (44%) 219

How often I need to receive it 45 (47%) 198

How soon I can receive it 49 (52%) 190

#### Is there any other additional information on CAR T-Cell therapy?

responses / none-responses / total surveyed 109 / 216 / 325

Yes 32 (29%)

No 77 (71%)

*Note:*

Ranking Score is the sum of the inverse rank order by the respondent then summed across the sample. Respondents did not need to rank all question options.

#### Other (open responses)

responses: 32

## Q17. Based on the information you have so far on bispecific antibodies, if it’s available to you, how likely would you like to try it for your multiple myeloma?

#### (Select all that apply)

responses / none-responses / total surveyed 198 / 127 / 325

**Responses**

Very likely 91 (46%)

Likely 55 (28%)

Neutral 22 (11%)

Unlikely 3 (2%)

I need more information to decide 26 (13%)

I have not heard of a bispecific antibody 1 (1%)

Note: No respondents selected: “Very Unlikely” or “I have already received one.”

## Q18. What additional information on bispecific antibodies do you need to support your decision making for your multiple myeloma treatment?

#### (Rank from the most to the least important, ranking all is not required)

n (%) Ranking Score

responses / none-responses / total surveyed 76 / 249 / 325

#### Responses

Side effects 56 (74%) 279

Am I the right patient to receive it 48 (63%) 246

Efficacy – how well the therapy will provide me the desired clinical outcome 68 (89%) 390

What is the administration process and procedure 41 (54%) 206

Costs to me 41 (54%) 196

How soon I can receive it 37 (49%) 177

How often I need to receive it 38 (50%) 157

Where can I receive it 38 (50%) 155

How will this therapy impact my family or caregivers 31 (41%) 120

#### Is there any other additional information on bispecific antibodies?

responses / none-responses / total surveyed 96 / 229 / 325

Yes 21 (22%)

No 75 (78%)

*Note:*

Ranking Score is the sum of the inverse rank order by the respondent then summed across the sample. Respondents did not need to rank all question options.

#### Other (open responses)

responses: 20

**Table S3.** Impact of disease status and prior treatment on patient multiple myeloma treatment decision-making

| **Patient involvement in MM treatment decision-making** | | | | | | |
| --- | --- | --- | --- | --- | --- | --- |
|  | Total | NDMM | RRMM | 1-3 LOT | ≥ 4 LOT | TEC plus ≥ 4 LOT |
| Respondents (N) | 323 | 71 | 147 | 86 | 61 | 55 |
| Very involved, I drive the conversation | 102 (32%) | 27 (38%) | 47 (32%) | 28 (33%) | 19 (31%) | 16 (29%) |
| Very involved, my doctor drives the conversation but considers my goals and preferences with recommendations | 190 (59%) | 33 (46%) | 93 (63%) | 54 (63%) | 39 (64%) | 36 (65%) |
| Somewhat involved, my doctor makes the decision and asks for my agreement | 30 (9%) | 11 (15%) | 6 (4%) | 3 (3%) | 3 (5%) | 3 (5%) |
| Not so involved, I let my doctor make the decision | 1 (<1%) | - | 1 (1%) | 1 (<1%) | - | - |
| **Statistical comparisons** | | | | | | |
| Difference from NDMM (*p* value) | - | - | 0.027 | NS | NS | NS |
| Difference from 1-3 LOT (*p* value) | - | - | - | - | NS | NS |

Note: Statistical comparisons performed with a Chi Square analysis across selection options, *p* values were adjusted using the Benjamini-Hochberg’s method for 6 multiple comparisons.

Abbreviation: NS, not significant
